# microRNA-214 promotes epithelial-mesenchymal transition and metastasis in lung adenocarcinoma by targeting the suppressor-of-fused protein (Sufu)

**DOI:** 10.18632/oncotarget.5478

**Published:** 2015-10-08

**Authors:** Haixia Long, Zhongyu Wang, Junying Chen, Tong Xiang, Qijing Li, Xinwei Diao, Bo Zhu

**Affiliations:** ^1^ Institute of Cancer, Xinqiao Hospital, Third Military Medical University, Chongqing, China; ^2^ Department of Immunology, Duke University Medical Center, Durham, North Carolina, USA; ^3^ Department of Pathology, Xinqiao Hospital, Third Military Medical University, Chongqing, China; ^4^ Biomedical Analysis Center, Third Military Medical University, Chongqing, China

**Keywords:** miR-214, metastasis, EMT, lung adenocarcinoma, suppressor-of-fused protein

## Abstract

Distant metastasis is the major cause of cancer-related deaths in patients with lung adenocarcinoma (LAD). Emerging evidence reveals that miRNA is critical for tumor metastasis. miR-214 expression has been associated with LAD progression. However, whether and how miR-214 is involved in the development and metastasis of LAD remain unaddressed. Here, we found that the expression of miR-214 was elevated in LAD and correlated positively with LAD metastasis and epithelial-mesenchymal transition (EMT). In addition, we found that miR-214 enhanced the molecular program controlling the EMT of LAD cells and promoted LAD cell metastasis both *in vitro* and *in vivo*. This study thus provides the first evidence to show that the miR-214 expression by LAD cells contributes to the EMT and metastasis of LAD. Mechanistically, Sufu was identified as an important miR-214 functional target for the EMT and metastasis of LAD, ectopic expression of Sufu alleviated miR-214 promoted EMT and metastasis. Importantly, the expression of Sufu inversely correlated with the expression of miR-214 and vimentin and positively associated with the expression of E-cadherin in the tumor cells from human LAD patients. Collectively, this study uncovers a previously unappreciated miR-214-Sufu pathway in controlling EMT and metastasis of LAD and suggests that interfering with miR-214 and Sufu could be a viable approach to treat late stage metastatic LAD patients.

## INTRODUCTION

Lung cancer is the most common cancer worldwide, accounting for 1.3 million deaths annually. Lung adenocarcinoma (LAD) is the most common form, which comprises nearly 40% of lung cancers. Approximately 90% of LAD patients result in distant metastasis at the advanced stage [1]. A better understanding of the molecular mechanisms underlying distant metastasis is required to facilitate the development of effective therapeutic strategies for LAD patients.

The EMT (epithelial-mesenchymal transition) is an evolutionarily conserved development process during which epithelial cells lose polarity and develop a mesenchymal phenotype, and this transition has been implicated in the initiation of metastasis [2, 3, 4]. In the tumor microenvironment, the EMT can be triggered by many signaling pathways, including transforming growth factor-β (TGF-β) [5, 6], epidermal growth factor [7], fibroblast growth factor [8], matrix metalloproteinase [9], hypoxia [6, 10]. Among these signaling pathways, TGF-β and hypoxia are the best characterized and most frequently used inducers of the EMT.

MicroRNAs (miRNAs) are central to tumor metastasis. It is therefore important to reveal what miRNA(s) and how they control tumor metastasis [11]. Recently, the correlation of miRNA dysregulation with the EMT or metastasis has been increasingly reported. For instance, the miR-200 family members (miR-200a, b and c, miR-141 and miR-429) inhibit the EMT by targeting the repressors of E-cadherin that induce epithelial differentiation, or by targeting the EMT activators known as ZEB1/2 genes [12–14]. miR-194 was reported to correlate inversely with the mesenchymal marker N-cadherin [15]. miR-448 suppression reportedly induces EMT by promoting SATB1 expression, leading to increased twist expression [16]. Although some miRNAs function as metastasis promoters, miR-103/107 over-expression is sufficient for inducing EMT *in vitro* and also induces pro-metastasis in mammary metastasis models [17]. miR-22 has been found to trigger the EMT, and it promotes metastasis by inhibiting the demethylation of the miR-200 promoter [12–18]. These data suggest that miRNAs dysregulation may play an important role in tumor metastasis.

Previously, miR-214 has reportedly been aberrantly expressed in different types of tumors. It plays a dual role by regulating tumor growth according to the tumor types, as oncogenes or tumor suppressors. On the one hand, miR-214 is highly expressed in melanoma and promotes tumor cell migration by up-regulating ALCAM and miR-148b down-regulation [19]. On the other hand, miR-214 can also suppress tumor development, and its expression is correlated with poor clinical outcomes in hepatocellular carcinoma, promoting apoptosis and angiogenesis by suppressing HDGF [20]. In LAD, miR-214 expression was significantly higher than it was in normal tissue [21] and was associated with advanced tumor stage, poor overall survival and higher recurrence rates [22–23], which suggest that miR-214 is important for LAD development. However, none of the previous studies have systematically investigated the role of miR-214 in the development of metastatic disease in LAD.

In this study, we demonstrated the function of miR-214 in LAD and found that miR-214 strongly activates the EMT, and it ultimately promotes LAD metastasis by targeting suppressor-of-fused (Sufu), a negative regulator of the Hedgehog signaling pathway. These observations suggest that miR-214 can be a therapeutic target for preventing LAD metastasis.

## RESULTS

### miR-214 is increased in LAD and positively associated with metastasis

**Figure 1:**
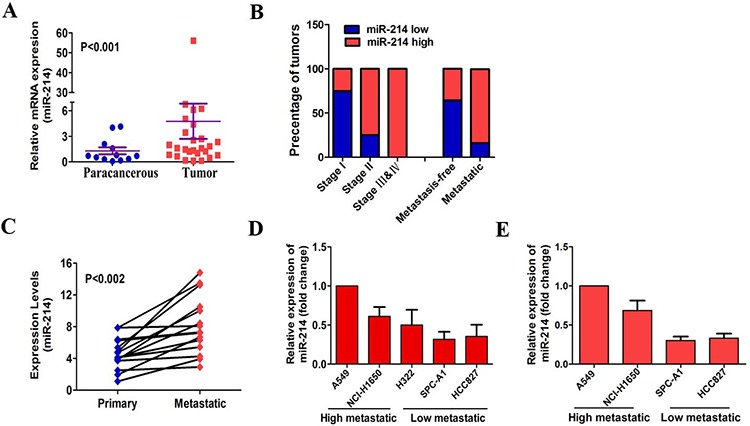
miR-214 is up-regulated in LAD and positively associated with metastasis **A.** The quantitation of miR-214 expression in human primary LAD cancer tissues (*n* = 22) and paracancerous tissues (*n* = 13). U6 was used as a loading control for miR-214. The relative expression levels were normalized to the mean value of all patients. **B.** The correlation between the miR-214 levels and clinical pathological parameters in LAD patients. **C.** miR-214 levels in primary lung tumors and matched lymph node metastases by real-time PCR (*n* = 15). **D.** miR-214 expression was detected among five LAD cell lines in a descending order of metastatic potential. **E.** 1 × 10^6^ SPC-A1, HCC827, A549 or NCI-H1650 cells were simultaneously injected into the right flank of SCID mice. Primary tumors were collected to investigate miR-214 expression levels by qRT-PCR (*n* = 6). All the experiments were performed at least three times and the data are expressed as the means ± SD. The statistical significance of differences was measured by unpaired student's *t*-test (A) and paired student's *t*-test (C).

To demonstrate the miR-214 expression in LAD, we first examined the miR-214 expression levels in 22 primary and 13 para-cancerous LAD tissues using quantitative real-time PCR (qRT-PCR). Our results indicated that the miR-214 expression was significantly higher in tumor tissues compared with paracancerous tissues (*p* < 0.001, Figure 1A), which is consistent with previous reports [21–23]. To understand the potential roles of miR-214 in LAD, we analyzed the correlation between the miR-214 levels and the clinical pathological parameters in LAD patients. We found that almost all (100%) LAD patients with advanced stage III &IV cancer showed high miR-214 expression, whereas those with early stage I (75%) showed low miR-214 (Figure 1B). Most of the tumors with metastases (83.3%) exhibited high miR-214 expression (only 16.7% showed low miR-214 expression). Inversely, most of the tumors from metastasis-free patients (64.3%) showed low miR-214 expression (Figure 1B). To confirm the correlation between miR-214 and metastasis, we compared the miR-214 expression levels in primary tumors with their matched metastatic tissues in 15 LAD patients. We found that the miR-214 expression was significantly higher in metastatic tumors compared with the matched primary tumors (*p* < 0.002, Figure 1C). Meanwhile, we tested the miR-214 levels in five LAD cell lines (A549, NCI-H1650, H322, SPC-A1 and HCC827) with different metastatic potentials [24, 25]. Among the five LAD cell lines with a descending order of metastatic potentials, their endogenous level of miR-214 was correspondingly decreased (Figure 1D). Moreover, *in vivo*, tumors derived from lower metastatic potential cells (SPC-A1, HCC827) expressed lower levels of miR-214 than that the tumors derived from higher metastatic potential cells (A549 and NCI-H1650) (Figure 1E), suggesting that the metastatic potential was positively associated with endogenous miR-214 levels. Collectively, our findings indicate a strong positive correlation between miR-214 and LAD metastasis.

### miR-214 enhances LAD cell migration and invasion *in vitro* and promotes their metastasis *in vivo*

**Figure 2:**
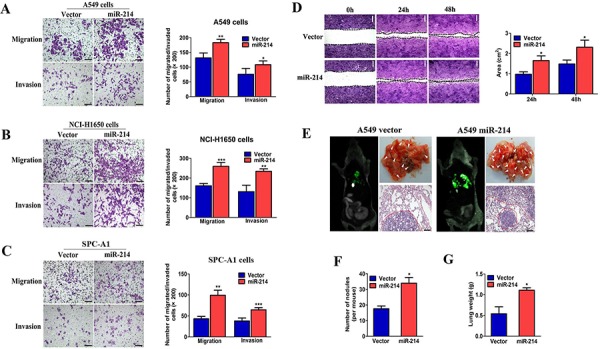
miR-214 enhances LAD cell migration and invasion *in vitro* and promotes their metastasis *in vivo* **A, B.** and **C.** The migration and invasion abilities of A549 (A), NCI-H1650 (B) and SPC-A1 cells (C) when transfected with the miR-214 over-expression vector or an empty vector as assessed by Transwell migration and Matrigel invasion assay for 24 hours. **D.** A wound-healing assay performed with miR-214-over-expressed A549 cells at 0, 24, and 48 hours, and the average wound-healing area. **E.** Images showing the lung metastatic foci in recipient mice on day 30 after vein injection and hematoxylin and eosin (H&E) staining (*n* = 10). **F–G.** The quantitation of metastatic nodes and weights in the lungs of recipient mice (*n* = 10). All experiments were performed at least three times, and the data are expressed as means ± SD. The statistical significance of differences was measured by unpaired student's *t*-test. *P < 0.05, **P < 0.01, ***P < 0.001.

As described above, we found that miR-214 was positively associated with LAD metastasis. In view of these findings, we focused on miR-214 for further metastatic functional studies. We first generated LAD cell lines with stable miR-214 over-expression (A549 and NCI-H1650) using lentivirus transfection. Immunofluorescence (Supplementary Figure S1A) and qRT-PCR analysis (Supplementary Figure S1B) demonstrated that NCI-H1650, A549 and SPC-A1 cells successfully over-expressed miR-214. Next, we performed a Boyden chamber migration/invasion and scratch wound-healing assay for the metastatic function study *in vitro*. We found that the ectopic expression of miR-214 dramatically increased the migratory and invasive abilities of both miR-214 high expressing cells (NCI-H1650 and A549 cells) and miR-214 low expressing cells (SPC-A1) (Figure 2A–2C). In addition, miR-214 over-expressed A549 cells migrated more rapidly to close the scratched wounds, compared with cells with the control vector, as tested by using a scratched wound-healing assay (Figure 2D). *In vivo* xenograft experiments showed that miR-214-over-expressing A549 cells displayed more visible metastatic nodules in the lungs compared with those from mice that were carrying the vector at 30 days after tail vein injection (Figure 2E–2G, *n* = 10). Collectively, our data suggested that miR-214 overexpression significantly enhanced the migratory and invasive abilities of LAD cells *in vitro* and markedly promoted LAD metastasis *in vivo*.

### miR-214 promoted LAD metastasis is mediated by the EMT

The EMT is one of the key initiation steps in the metastasis process, which provides cancer cells with motility, invasion and migration properties [2, 3, 4]. Therefore, we further investigated whether miR-214-promoted LAD metastasis is mediated by the EMT process. Because TGF-β and hypoxia are the best characterized and most often used as inducers of EMT [6, 10], we first established a hypoxia-induced EMT model in three LAD cell lines (A549, NCI-H1650 and SPC-A1) to study the miR-214 function in the EMT. After culturing the cell lines under normoxia (21% O_2_) or hypoxia (0.5% O_2_) for 24 hours, western blots (Supplementary Figure S2A) and qRT-PCR analysis (Supplementary Figure S2B) showed that three cell lines resulted in a significant loss of E-cadherin and an increase in vimentin after being exposed to hypoxia. We then detected the miR-214 expression level in these three cell lines after hypoxia exposure. As shown in Supplementary Figure S3A, the miR-214 expression exhibited a 2 ~ 5-fold increase in all three LAD cells under hypoxic conditions than under normoxia. The above results were also consistently repeated both in the TGF-β-induced EMT model and the paclitaxel-induced EMT model [26] (Supplementary Figure S2C–S2D, Supplementary Figure S3B and S3C). To compare the different expressions between epithelial cells and mesenchymal cells, we used E-cadherin^low^ as the marker for mesenchymal cells and E-cadherin^high^ as the marker for epithelia to sort out these two populations of cells for both A549 and NCI-H1650 cells after hypoxia exposure. As shown in Supplementary Figure S3D, the miR-214 expression levels were much higher in E-cadherin^low^ cells than E-cadherin^high^ cells in both A549 and NCI-H1650 cells. These results showed that miR-214 was increased in the EMT cells of LAD, suggesting that miR-214 might activate the EMT process in LAD.

**Figure 3:**
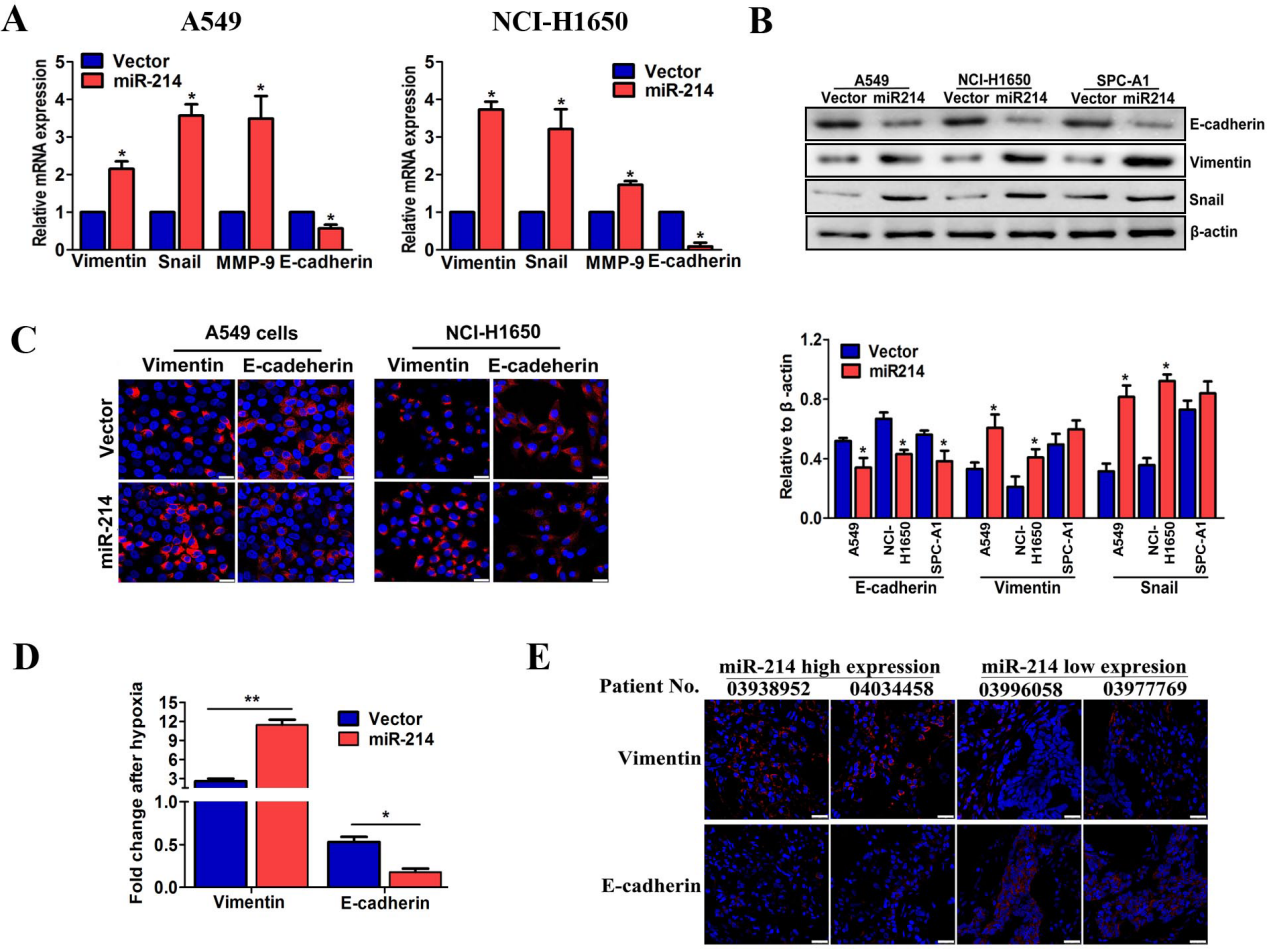
miR-214-promoted LAD metastasis is mediated by the EMT **A.** The expression of EMT markers as analyzed in miR-214-over-expressed A549 cells and NCI-H1650 by real-time PCR. **B.** EMT markers in three miR-214 over-expressed LAD cell lines as analyzed by western blots. **C.** The expression of EMT markers was analyzed by immunofluorescent staining in miR-214-over-expressed A549 cells and NCI-H1650. The nuclei were stained with DAPI. Scale bar = 25 μm. **D.** The expression of EMT markers in miR-214-over-expression A549 cells under hypoxia treatment were quantitated by real-time PCR. **E.** An immunofluorescence analysis of vimentin and E-cadherin expression in patients with lower (*n* = 6) and higher miR-214 expression levels (*n* = 6). The nuclei were staining with DAPI. Scale bar = 25 μm. All experiments were performed at least three times and the data are expressed as means ± SD. The statistical significance of differences was measured by unpaired student's *t*-test. *P < 0.05, **P < 0.01.

To confirm this hypothesis, we first used miR-214-over-expressing A549 and NCI-H1650 cells to analyze the EMT hallmark changes in both the mRNA and protein levels. As shown in Figure 3A–3C, the epithelial marker E-cadherin was significantly decreased after miR-214 overexpression. By contrast, the mesenchymal markers vimentin, MMP-9 and snail were increased in miR-214 over-expressed cells compared with the vector cells. Furthermore, under hypoxic conditions, the miR-214 over-expressing cells had more decreased E-cadherin and increased vimentin compared with the vector control cells (Figure 3D). More importantly, tumor tissues from LAD patients with high miR-214 expression showed high levels of vimentin and low levels of E-cadherin, whereas the reverse results were shown in miR-214 low expression groups (Figure 3E, *n* = 6 paired). Taken together, these results demonstrate that miR-214 enhances the EMT process in LAD cells.

**Figure 4:**
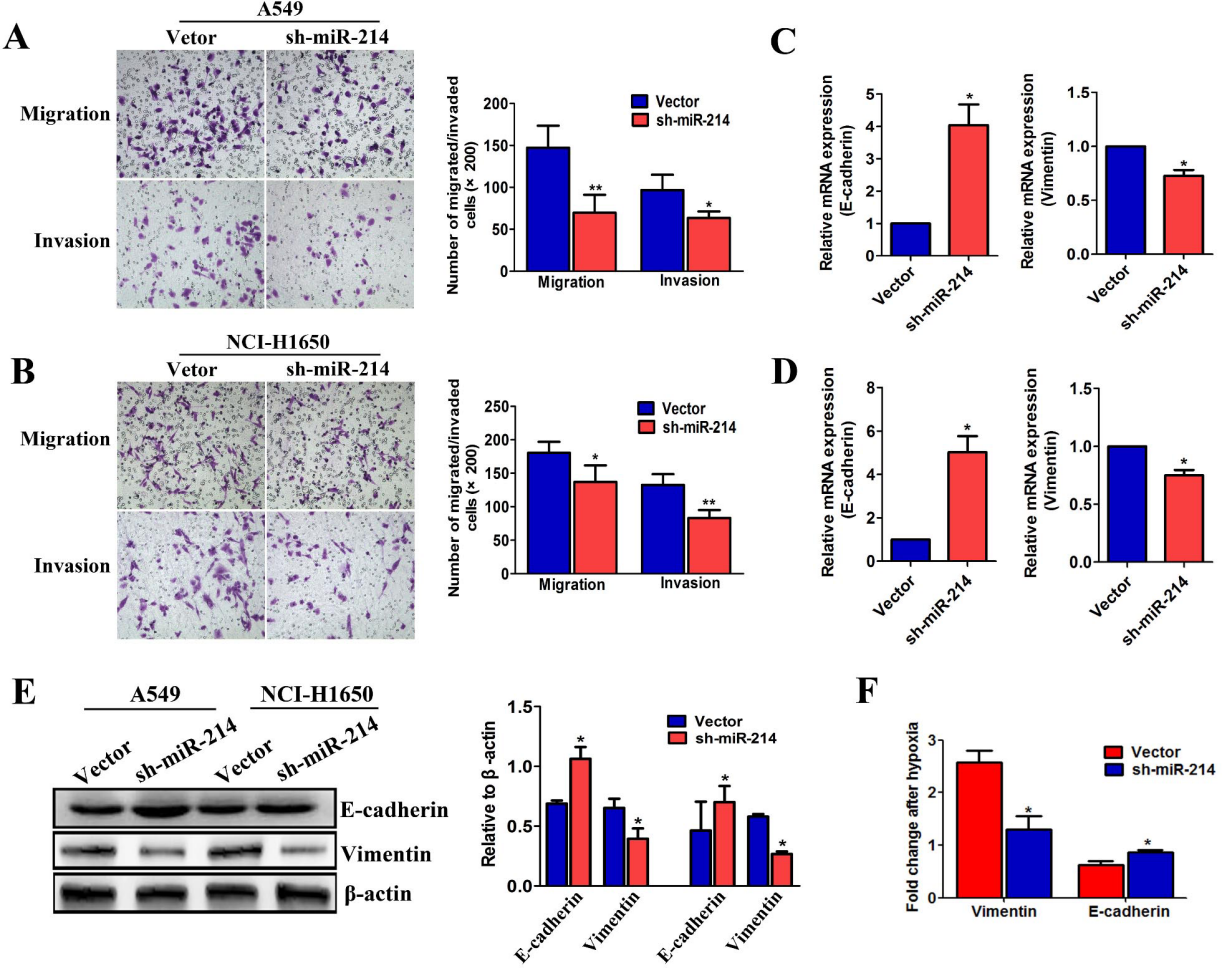
The down-regulation of miR-214 suppresses the EMT and migration ability in LAD cells **A.** and **B.** The migration and invasion abilities of A549 (A) and NCI-H1650 cells (B) as transfected with the sh-miR-214 vector or an empty vector and assessed by Transwell migration and Matrigel invasion assay for 24 hours. **C–D.** The expression of EMT markers analyzed in sh-miR-214-transfected A549 cells (C) and NCI-H1650 (D) by real-time PCR. **E.** EMT markers in sh-miR-214-transfected A549 and NCI-H1650 cells and analyzed by western blotting. **F.** The expression of EMT markers in sh-miR-214-transfected A549 cells under hypoxia treatment were quantitated by real-time PCR. All experiments were performed at least three times and the data are expressed as the means ± SD. The statistical significance of differences was measured by unpaired student's *t*-test. *P < 0.05, **P < 0.01.

Having shown that miR-214 overexpression could enhance the EMT process in LAD cells, we next employed a loss-of-function approach by using shRNA (Supplementary Figure S1C) to investigate its role in the EMT process. As anticipated, the migratory and invasive capabilities of both A549 and NCI-H1650 cells were significantly reduced by miR-214 inhibition (Figure 4A–4B). In addition, as shown in Figure 4C–4E, the epithelial marker E-cadherin was increased, and the mesenchymal marker vimentin was decreased in sh-miR-214-transfected A549 and NCI-H1650 cells, compared with the vector groups. Furthermore, the sh-miR-214-transfected A549 cells showed less E-cadherin and vimentin changes compared with the control cells under hypoxic conditions (Figure 4F). Collectively, our findings suggest that miR-214-promoted LAD metastasis is mediated by the EMT.

### Sufu is a direct target of miR-214 in LAD cells

**Figure 5:**
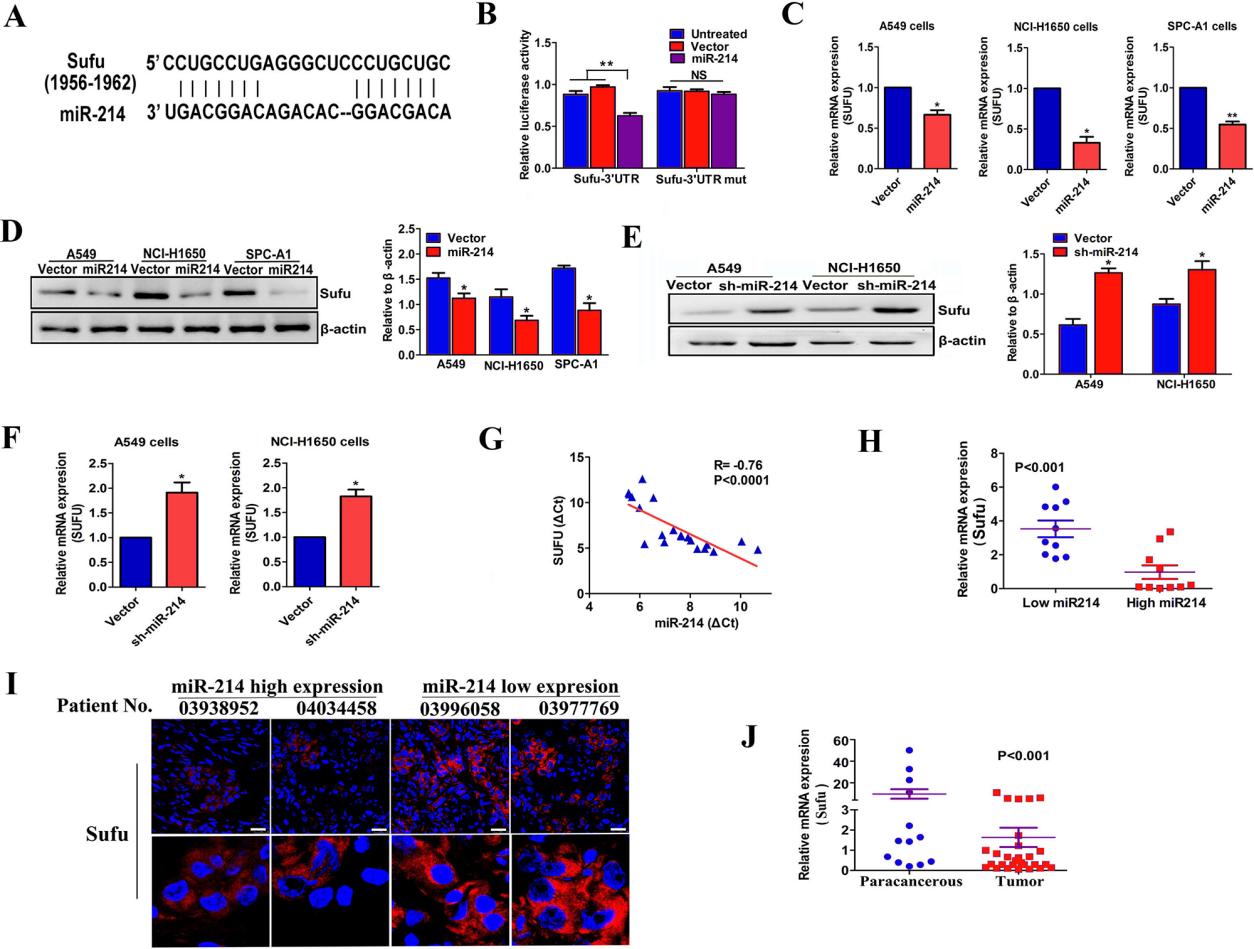
Sufu is a direct target of miR-214 in LAD cells **A.** A schematic representation of putative miR-214 binding in the 3′ UTR of Sufu at 1956–1962 nt. **B.** Dual-luciferase assays showing the repression of wild-type UTR (Sufu-3′UTR) or mutant UTR (Sufu-3′UTR mut) following the transfection of the vector or miR-214 over-expression vector. **C–D.** The inverse correlation between miR-214 and Sufu expression in miR-214 over-expression cells were determined by real-time PCR (C) and western blots (D) in three LAD cells (A549, NCI-H1650, and SPC-A1). **E–F.** Similar to B–C, the Sufu levels were detected by real-time PCR and western blots in miR-214-silenced cells. **G.** The inverse correlation between miR-214 and Sufu expression in 20 clinical samples was determined by Pearson's correlation coefficient (*R* = −0.76, *p* < 0.0001). **H–I.** The relative level of Sufu in LAD patients with lower or higher miR-214 expression as tested by real-time PCR and immunofluorescence. **J.** The quantitation of Sufu expression in primary human LAD tissues (*n* = 22) and paracancerous tissues (*n* = 13). β-actin was used as a loading control for Sufu. The relative expression levels were normalized to the mean values of all patients. All experiments were performed at least three times and the data are expressed as means ± SD. The statistical significance of differences was measured by unpaired student's *t*-test. The Pearson's correlation coefficient was used to assess the correlation between miR-214 and Sufu level (G) *P < 0.05, **P < 0.01, ns, not significant.

To investigate the roles for the target gene of miR-214 in the EMT process and metastasis in LAD, we first performed a miRNA target gene prediction with miRanda and Targetscan databases. We found that Sufu exhibited miR-214-binding sequences in its 3′-UTR regions (nucleotides 1956–1962, Figure 5A). Luciferase assays were performed to obtain direct evidence that Sufu is a target of miR-214. As expected, the luciferase activity was decreased by miR-214 over-expression when the Luc-Sufu-wt was present, compared with the luciferase activity in the Luc-Sufu-mu, suggesting that miR-214 reduced the luciferase activity of Luc-Sufu-wt but had no effect on Luc-Sufu-mu (Figure 5B). To confirm that Sufu acts as a miR-214 target, we examined the Sufu expression in miR-214 knockdown or over-expression LAD cells. The Sufu expression was decreased at both the mRNA (Figure 5C) and protein levels (Figure 5D) in A549, NCI-H1650 and SPC-A1 cells after being transfected with miR-214, compared with the vector transfection. Reciprocally, the miR-214 knockdown was accompanied by an increase in the Sufu expression in A549 and NCI-H1650 cells (Figure 5E–5F). More importantly, an inverse correlation was found between miR-214 expression and Sufu expression in 18 cases of clinical LAD tissues (*p* < 0.0001, *R* = −0.76, Figure 5G). Tumors with a high level of miR-214 expression showed lower Sufu expression, whereas the reverse results were shown in low miR-214 expression groups (*p* < 0.001, Figure 5H). These results were also demonstrated by immunofluorescence for Sufu expression (Figure 5I). Furthermore, the Sufu expression in primary tumor tissues is lower than that of para-cancerous tissues (*p* < 0.001, Figure 5J). Taken together, our results suggest that miR-214 promotes LAD metastasis by directly targeting Sufu.

### Sufu was a direct functional target of miR-214 in LAD metastasis

**Figure 6:**
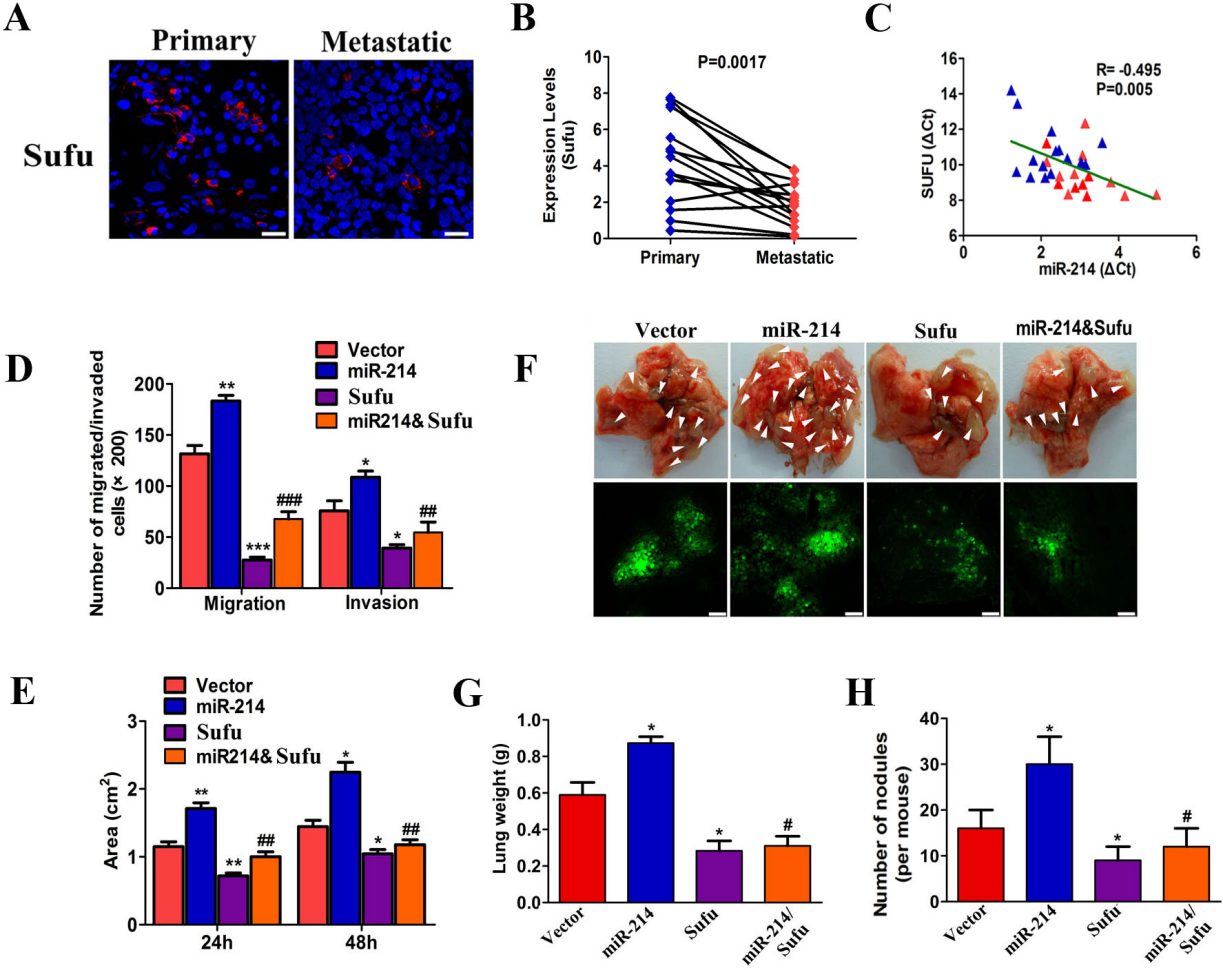
Sufu was a direct functional target of miR-214 in LAD metastasis **A.** Microscopic images showing Sufu protein levels in primary LAD and matched metastasis by immunofluorescence (*n* = 10 paired). The nuclei were stained with DAPI. Scale bar = 25 μm. **B.** Sufu expression levels in primary LAD and matched metastasis by real-time PCR (*n* = 15, *P* = 0.0017). **C.** The correlation between miR-214 and Sufu expression in primary and matched metastasis (*n* = 30, *P* = 0.005, *R* = −0.495). **D.** The invasive and migration capacities of A549 cells after the indicated treatment as assessed by Transwell migration assay and Matrigel invasion assay. **E.** Wound-healing assay images of A549 cells after the indicated treatments at 0, 24, and 48 hours; the average wound-healing area is presented. **F.** Images showing lung metastatic foci in recipient mice on day 30 after vein injection (*n* = 6). **G–H.** The quantitation of metastatic nodes and lung weights in recipient mice (*n* = 6). All experiments were performed at least three times, and the data are expressed as means ± SD. The statistical significance of differences was measured by unpaired student's *t*-test (D,E,G,H) and paired student's *t*-test (B). The Pearson's correlation coefficient was used to assess the correlation between miR-214 and Sufu level (C) *means compared with the vector group, *P < 0.05, **P < 0.01, ***P < 0.001. #means compared with the miR-214 overexpression group. ^#^*P* < 0.05, ^##^*P* < 0.01, ^###^*P* < 0.001.

Sufu is a negative regulator of the Hedgehog signaling pathway, interacting directly with the Gli family of transcription factors [25, 27]. The Hedgehog/Gli pathway is regarded as a proto-oncogene and harbors migration-promoting activity [28–30]. These findings suggest that Sufu might be a regulator for tumor metastasis. To determine the potential function of Sufu in LAD metastasis, we investigated the expression levels of Sufu in six primary LADs, and 15 matched with metastasis according to immunofluorescence and qRT-PCR. The results showed a significant decrease in the Sufu expression of metastatic tissues compared with matched primary tumors (Figure 6A–6B, *P* < 0.0017), which are consistent with the E-cadherin and vimentin expression panels in primary and metastatic tumors (Supplementary Figure S4). Furthermore, there was also an inverse relation between the Sufu and miR-214 expression levels in primary and metastatic tissues (*P* < 0.005, *R* = −0.495, Figure 6C). These results provide more evidence supporting the potential inhibition of Sufu in LAD metastasis.

To test whether Sufu is functionally regulated by miR-214 in LAD metastasis, we generated stable Sufu and miR-214 and Sufu-over-expressing A549 cells (Supplementary Figure S5). We observed that Sufu over-expression significantly decreased A549 cell migration and invasion *in vitro*, whereas miR-214 over-expression in A549 cells led to a dramatic increase in migration and invasion (Figure 6D–6E, Supplementary Figure S6). Interestingly, this over-expression of miR-214-associated phenotype changes could be partially rescued by Sufu over-expression (Figure 6D–6E, Supplementary Figure S6). In the *in vivo* xenograft mouse model, we found that the lungs from the mice carrying miR-214 over-expressing A549 cells displayed more visible metastatic nodules than the vector control, and the Sufu over-expressing A549 cells displayed less visible metastatic nodules than the vector control. Consistent with the *in vitro* results, the over-expression of Sufu could partially abolish the pro-metastatic roles of miR-214 over-expression (Figure 6F–6H, *n* = 6). Collectively, all these results demonstrated that miR-214 promotes LAD metastasis by targeting Sufu.

### The miR-214-mediated EMT process in LAD cells depends on Sufu inhibition

**Figure 7:**
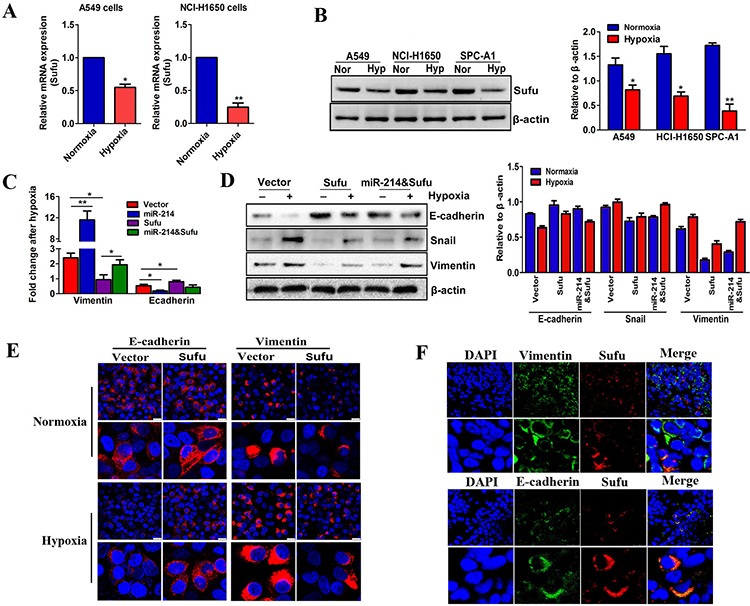
The miR-214-mediated EMT process in LAD cells depends on Sufu inhibition **A–B.** Real-time PCR (A) and western blot (B) analyses of Sufu expression in three LAD cells under normoxia (21% O_2_) or hypoxia (0.5% O_2_) treatment for 24 hours. β-actin was used as an internal loading control. **C–D.** The fold changes in the EMT markers detected by real-time PCR or western blots in Sufu-over-expressed or Sufu and miR-214-co-overexpressed A549 cells. **E.** The immunofluorescence detection of EMT markers changed in Sufu-over-expressed cells under normoxia or hypoxia treatment. Scale bar = 25 μm. **F.** Confocal microcopy of LAD patient tissues co-stained for E-cadherin, or vimentin with Sufu. The nuclei were staining with DAPI. Scale bar = 25 μm. All experiments were performed at least three times, and the data are expressed as means ± SD. The statistical significance of differences was measured by unpaired student's *t*-test. *P < 0.05, **P < 0.01.

To determine the role of Sufu in the EMT process of LAD cells, Sufu expression was examined and compared under normoxic and hypoxic conditions by qRT-PCR and western blotting. We found that the Sufu expression in both A549 and NCI-H1650 cells was significantly decreased after exposure to hypoxia (Figure 7A–7B), which is consistent with the results in which miR-214 was increased by hypoxia (Supplementary Figure S3A). To test whether the miR-214-enhanced EMT process is dependent on Sufu inhibition, we used hypoxia as the EMT induction model and compared EMT-associated changes after hypoxia treatment among miR-214 overexpression, Sufu overexpression, Sufu&miR-214 overexpression and vector-transfected A549 cell groups. As shown in Figure 7C–7E, miR-214 over-expression caused EMT-related marker changes in the A549 cell line, whereas co-transfection with a Sufu over-expression vector partially abolished these changes at both the mRNA and protein levels. These findings suggest that miR-214 regulates the EMT by directly suppressing Sufu expression. Moreover, we used immunofluorescence to co-stain Sufu with E-cadherin or vimentin in LAD tissue sections to investigate the relations between Sufu expression and the epithelial and mesenchymal cell phenotypes at the single cell level *in situ*. Interestingly, the Sufu expressions were mostly co-stained with E-cadherin, and there were few cells with Sufu and vimentin co-staining (Figure 7F). These observations indicate that the miR-214-induced EMT process is dependent on Sufu inhibition.

## DISCUSSION

Emerging evidence suggest that the dysregulation of miRNAs contributes to tumor metastasis. However, limited miRNAs have been identified in the regulation of LAD metastasis. The present study identified miR-214 to promote LAD metastasis. For the first time, we revealed a positive correlation between miR-214 expression and LAD metastasis, providing novel insights into how miR-214 correlates with advanced tumor stages, poor overall survival and higher recurrence rates in lung cancer [22–23]. We demonstrated that miR-214 enhanced the migratory and invasive abilities of LAD cells *in vitro* with three different cell lines and with both gain-of-function and loss-of-function experiments and that it promoted A549 cell-bearing tumor metastasis in a xenograft mouse model. This finding is consistent with a previous report showing that miR-214 is highly expressed in melanoma and promotes tumor cell migration [19]. However, our results are in contrast to Salim's publication [31] in which miR-214 could decrease the invasiveness of non-small cell lung carcinoma (NSCLC) cells including U-1810 (lung squamous carcinoma) and H23 (LAD). In our study, we performed experiments on three different LAD cell lines and provided strong evidence with an *in vivo* xenograft mouse model and with clinical samples. Moreover, we demonstrated that miR-214 enhanced the EMT of LAD cell, an important process for metastasis, supporting miR-214 as the promoting function in LAD metastasis. Importantly, previous studies have shown that miR-214 expression has been associated with high grade and cancer progression in LAD [21–23], our studies not only confirmed that the expression of miR-214 was elevated in LAD, but also found that increased miR-124 expression correlated with LAD metastasis and epithelial-mesenchymal transition (EMT). However, this discrepancy might be related to the use of different cell lines (the H23 cell line *versus* the A549, NCI-H1650, and SPC-A1 cell lines) because the same miRNA could perform different functions through distinct pathways in a way that was dependent on the tissue or cell type [32].

The EMT process is a crucial step in initiating the metastatic spread of many tumor cells into distal organs in a variety of cancers [2–4]. Recently, miRNAs have been suggested to regulate EMT [12–18]. Here, we found miR-214 to be highly expressed in tumor cells that are proceeding with the EMT process accompanied by gaining mesenchymal markers and losing epithelial markers. Moreover, overexpression of miR-214 greatly promoted the EMT in LAD cells, and knocking down miR-214 by shRNA inhibited EMT. Importantly, in human LAD tumor tissues, miR-214 expression was significantly associated with EMT-related markers; a high expression level of miR-214 significantly associated with low E-cadherin expression and high vimentin expression, which supports the loss- and gain- of function studies performed in this report by using LAD tumor cell lines. This is the first time, to our knowledge, that miR-214 has been shown to enhance the EMT process in LAD and any cancer types.

It is well established that miRNAs perform their function by regulating the expression of a target gene. Therefore, we decided to identify the functional target gene for miR-214 that was involved in LAD metastasis regulation. We thought Sufu would be the functional target gene of miR-214 after we performed a prediction with miRanda and Targetscan databases. Moreover, we found that miR-214 directly bound with the 3′UTR region of Sufu and suppressed Sufu expression. In addition, the overexpression or knock-down of miR-214 in LAD cells could decrease or increase Sufu expression, respectively, which consistence with that miR-214 expression was inversely correlated with Sufu expression in tumors from the patients with LAD. Although, miR-214 influences the formation of slow muscle cell types by targeting Sufu in zebrafish had been reported [33]. But there was no direct evidence to show the relationship between miR-214 and Sufu in cancer cells. This is the first time to demonstrate that miR-214 directly regulates the Sufu signaling pathway in LAD cells.

The aberrant activation of the Hedgehog/Gli pathway has been implicated in tumor genesis and metastasis in various cancer types, including brain and breast, LAD [25–29]. Gli-1 is the most important transcription factor acting downstream of hedgehog signaling used as a Hedgehog signaling effector, which regulates the EMT regulators SIP1, ZEB2 and ZFHX1B [34–38]. In LAD, the overexpression of nuclear Gli-1 was positively correlated with lymph node metastases, implying a promoter role for activated Gli-1 in LAD progression and metastases [34–35]. Sufu was reported to be an essential intracellular and negative regulator of the Hedgehog pathway, which affected the production of Gli activators and repressors that were essential for graded Hh responses [25–29, 34–38]. Sufu reportedly functions as a tumor suppressor [39], the loss of its function causes excessive tumor cell proliferation [40] and angiogenesis [41]. However, there was no direct evidence to show the role of Sufu in tumor metastasis, not to mention its function in the EMT process of LAD. In this study, we illustrated the inhibitory effect of Sufu on metastasis and the EMT in LAD. In addition, Sufu over-expression can significantly rescue the miR-214 promoted EMT and metastasis. Most importantly, we found that the expression of Sufu inversely correlated with the expression of vimentin and positively associated with the expression of E-cadherin in the tumor cells from human LAD patients. Moreover, Sufu over-expression was associated with a significant export of Gli-1 from the nucleus to the cytoplasm. The Sufu promoted effects were inhibited when miR-214 was over-expressed (Supplementary Figure S7). This finding is consistent with a previous report that Sufu is a negative regulator of the Hedgehog signaling pathway, interacting directly with the Gli family of transcription factors [24–29]. The current study established a novel function of Sufu-miR-214 signaling axis in controlling EMT and metastasis of LAD.

We analyzed the relationship between Sufu expression level and survival rates of LAD from 200 samples, but there has no significantly correlation been observed (data not shown). (Gene expression data were obtained from the NCBI GEO datasets, GPL3877). This result can be explained by the following reasons: (1) It's well known that a single miRNA can regulate several (maybe even hundreds of) transcripts and a single transcript can have binding sites for (or can be regulated by) several miRNAs of the same or different sequence. (2) A single gene has complex functions; it can play a dual role by regulating tumor growth according to the specific situation, as oncogenes or tumor suppressors. It's no way to fully define the function of one gene. (3) Patients prognosis and survival could be affected by so many factors. Collectively, our study has significant implications for understanding the underlying mechanisms of how miR-214 contributes to tumor progress and poor clinical outcomes in LAD. miR-214 controls EMT and metastasis by directly silencing Sufu. The findings from this study suggest that interfering with miR-214 and Sufu could be a viable approach to treat late stage metastatic LAD patients.

## MATERIALS AND METHODS

### Cell culture and patient samples

Human LAD cell lines NCI-H1650, A549, SPC-A1, H322 and HCC827 were obtained from the American Type Culture Collection (ATCC). These cell lines were last tested and authenticated by short tandem repeat profiling in September, 2014. The cell lines were maintained in Dulbecco's modified Eagle's medium (DMEM) with 10% fetal bovine serum (FBS), 100 U/ml penicillin, and 100 μg/ml streptomycin, and they were incubated at 5% CO_2_ at 37°C. In experiments designed to induce EMT by hypoxia, the cells were cultured under normoxic conditions (21% O_2_) or hypoxic conditions (0.5% O_2_) for 24 hours, as previously detailed in the literature [6, 10]. To induce the EMT with TGF-β or paclitaxel, cells were treated with TGF-β (5 ng/ml) for 7 days [6] or with paclitaxel (5 ng/ml) for 2 days [26], and then the cells were collected for further study.

LAD tissues and their corresponding paracancerous tissues were collected by surgical resection at Daping Hospital (Chongqing, China) from Aug 2011 to Sep 2013. Fresh primary and metastatic LAD tissues were collected at Xinqiao hospital (Chongqing, China) from Aug 2012 to Sep 2014. The patient details are shown in Supplementary Table S1. This study was approved by the Institutional Review Board of the Third Military Medical University, and informed consent was obtained from each patient.

### RNA extraction and real-time PCR

miRNA expression levels were tested by using the qRT-PCR miRNA kit and qRT-PCR primer sets, according to the manufacturer's instructions (Ribobio, Guangzhou, China). Primers for human E-cadherin, snail, vimentin, matrix metalloproteinase-9 (MMP-9) and β-actin are listed in Supplementary Table S2. Real-time PCR was performed in an ABI7500 Prism Sequence Detection System (Applied Biosystems, Foster City, CA) by using a SYBR Green kit (TaKaRa, Tokyo, Japan), and the relative changes were quantified. Each experiment was repeated at least three times.

### RNA interference

LAD cells were stably infected with the pre-microRNA expression construct known as the lenti-miR expression plasmid, which contained the full-length miR-214 in H1-MCS-CMV-EGFP vector (GeneChem, Shanghai, China; vector information: http://www.genechem.com.cn/Zaiti.aspx?zt=GV259). The sh-miR-214 sequence (ACTGCCTGTCTGTGCCTGCTGT) was cloned into the H1-MCS-CMV-EGFP (GeneChem, Shanghai, China; vector information: http://www.genechem.com.cn/Zaiti.aspx?zt=GV159) to generate H1-MCS-CMV-EGFP-sh-miR-214 (GeneChem, Shanghai, China). For the overexpression of the Sufu gene, Sufu cDNA (lacking the miR-214 target site in the 3′UTR) was cloned into a lentiviral shuttle vector (GeneChem, Shanghai, China, (vector information: http://www.genechem.com.cn/Zaiti.aspx?zt=GV218). A non-targeting sequence was used as a lentivirus negative control and was purchased from Genechem (GeneChem, Shanghai, China). An infection efficiency >90% was verified by fluorescent microscopy and confirmed for miR-214 or Sufu expression.

### Migration and invasion assay

A migration assay was performed by using 24-well culture inserts with a porous polycarbonate membrane (8.0 μm, Millipore, Billerica, MA). For the Matrigel invasion assay, the filters were pre-coated with 30 μl of Matrigel (BD Biosciences, USA) for 3 hours. The migration and invasion assays were performed according to our previously published protocol [42]. In brief, 2 × 10^4^ cells in 200 μl of serum-free medium were added to the upper chamber, and 800 μl of medium with 5% serum was placed in the lower chamber. The plates were incubated for 24 hours at 37°C in 5% CO_2_. Cells that did not migrate or invade through the pores were removed with a cotton swab. Cells on the lower surface of the membrane were examined and counted under a microscope. Each experiment was repeated at least three times.

### Flow cytometry sorting

Dissociated LAD cells were counted and transferred to a 5 ml tube, washed twice with PBS, counted and re-suspended in PBS at 1 × 10^6^ cell/100 μl. These cells were then incubated with FITC-conjugated monoclonal antibody (mAb) specific to human E-cadherin (1:200, eBioscience, USA) or isotype-matched control mAb (1:200, eBioscience, USA) for 20 min on ice in the dark. After being washed twice with PBS, the samples were suspended in 500 μl of PBS. The cells were routinely sorted twice, and they were analyzed for E-cadherin purity, which was typically >97%. The data were analyzed by using CellQuest software.

### Immunofluorescence

An immunofluorescence analysis was performed on 8-μm-thick frozen sections, which were fixed with ice-cold 4% paraformaldehyde for 15 minutes and blocked with normal serum for 20 minutes at room temperature before being incubated with one or more specific antibodies against vimentin (1:200, Abcam, Cambridge), E-cadherin (1:200, Abcam, Cambridge), Sufu (1:200, Abcam, Cambridge) or Gli-1 (1:200, Abcam, Cambridge) overnight and in the dark at 4°C. After three washes, the slides were then stained with FITC-conjugated anti-rabbit antibodies or Cy3-conjugated anti-mouse antibodies (1:500, Abcam, Cambridge). The nuclei were counterstained with DAPI. Stained cells were visualized with an Olympus confocal microscope. All of the experiments were repeated at least three times.

### Western blot

All the cell lysates were prepared and western blot analyses were performed as previously described [42]. The following antibodies were used: E-cadherin (1:500, Abcam, Cambridge), snail (1:500, Abcam, Cambridge), vimentin (1:500, Abcam, Cambridge), Sufu (1:200, Santa Cruz Biotechnology, USA), twist (1:200, BD Biosciences, USA), and β-actin (1:400, Boster, China).

### 3′-UTR reporter luciferase assays

Under Lipofectamine-2000 (Invitrogen), the A549 cells were transfected with luciferase vectors (an empty luciferase vector, a luciferase vector containing the wild-type target gene's 3′-UTR, and a luciferase vector containing the mutant-type target gene's 3′-UTR) for Sufu, and together with miR-214 or the negative control. After 72 h, the luciferase activity was measured by using the Dual-Luciferase Reporter Assay System (Promega, Madison, USA). The data were presented as the ratios between firefly and Renilla fluorescence activities.

### *In vivo* xenograft experiments

Severe combined immunodeficient (SCID) mice were purchased from the Chinese Academy of Medical Sciences (Beijing, China). The mice were housed and maintained in laminar flow cabinets under specific pathogen-free conditions. For the xenograft experiments, A549 cells infected with lentivirus carrying a GFP-vector, miR214 and/or Sufu were injected into the tail veins. The organs of mice bearing tumors that stably expressed GFP were analyzed by using a bioluminescence imaging system [42]. The organs were harvested for further evaluation through real-time PCR, immunofluorescence, and H&E staining. Mouse care and use was performed in accordance with local ethical guidelines.

### Statistical analyses

All the data were presented as the means ± SD. The statistical analyses were performed by using Student's *t*-tests or one-way ANOVAs. A difference was considered to be statistically significant when *p* < 0.05. All of the statistical analyses were performed with SPSS 13.0 software.

## SUPPLEMENTARY FIGURES AND TABLES


