# Supplementary material for: microRNA-214 promotes epithelial-mesenchymal transition and metastasis in lung adenocarcinoma by targeting the suppressor-of-fused protein (Sufu)
Source: Oncotarget. 2015 Oct 8;6(36):38705–18. doi: 10.18632/oncotarget.5478 (PMC4770731; doi:10.18632/oncotarget.5478)
Supplement: Supplementary file 1 [file oncotarget-06-38705-s001.pdf]

SUPPLEMENTARY FIGURES AND TABLES

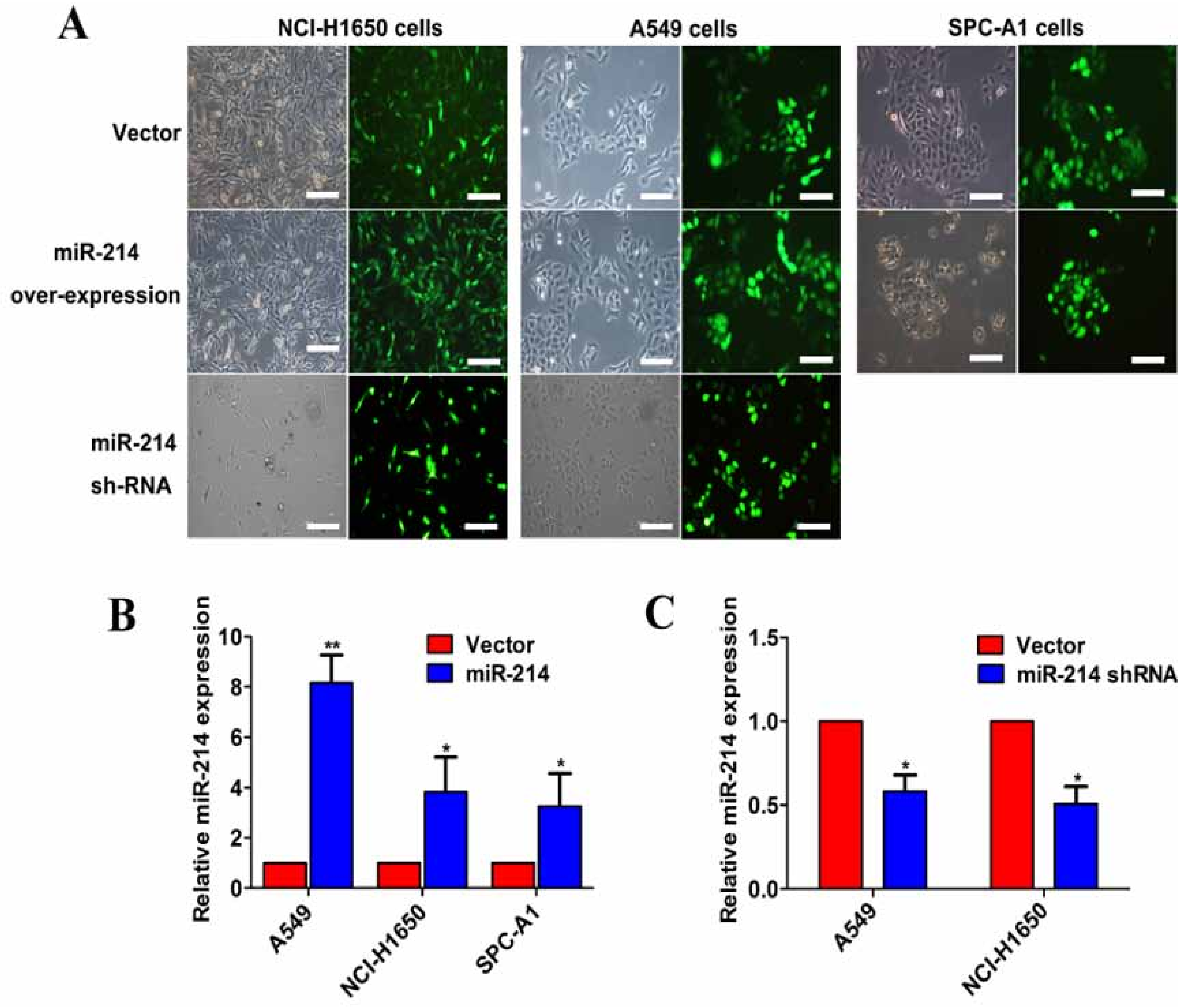

Supplementary Figure S1: A. A morphological image of A549, NCI-H1650 and SPC-A1 cells transfected with miR-214-overexpression, inhibitor or empty lentiviral vectors. **B–C.** miR-214 expression levels as tested by qRT-PCR in the indicated cells following transfection with the above-mentioned virus. All experiments were performed at least three times, and the data are expressed as means ± SD. The statistical significance of differences was measured by unpaired student's *t*-test. *$P < 0.05$, **$P < 0.01$.

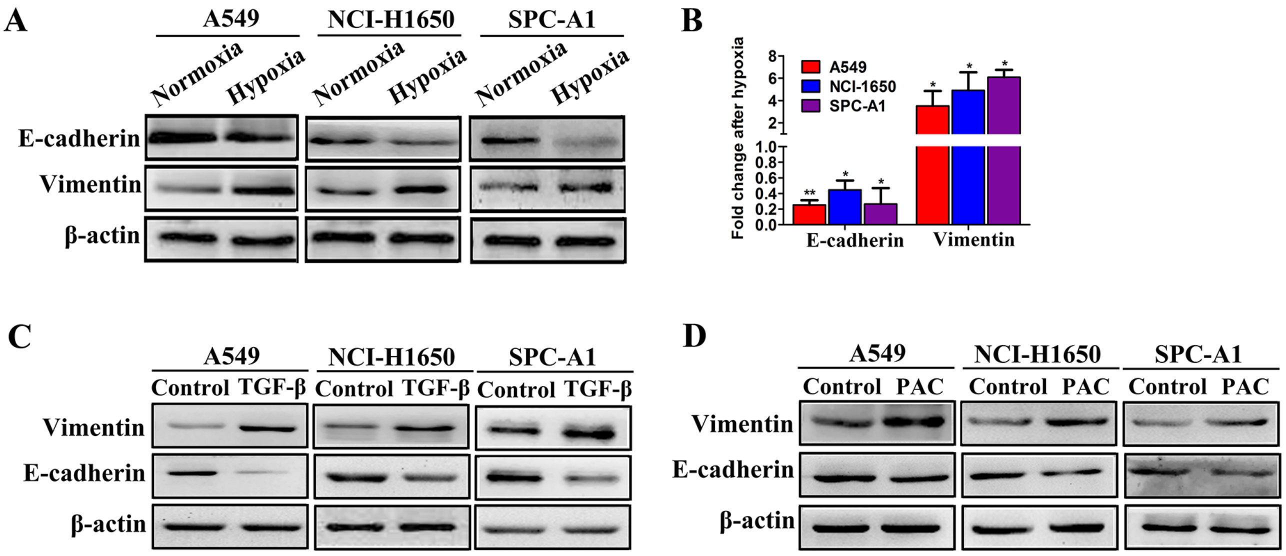

Supplementary Figure S2: A–B The expression of E-cadherin and vimentin under normoxia (21% O_2) or hypoxia (0.5% O_2) treatment for 24 hours in three LCCs (A549, NCI-H1650, and SPC-A1), as determined by western blots and real-time PCR. **C.** The TGF-β (5 ng/ml) treatment was performed for 7 days, and the EMT markers were analyzed by western blotting. **D.** The expressions of E-cadeherin and vimentin were detected by western blotting under paclitaxel (5 ng/ml) treatment for 2 days. All experiments were performed three times and the data are expressed as means ± SD. The statistical significance of differences was measured by unpaired student's *t*-test. *P < 0.05, **P < 0.01.

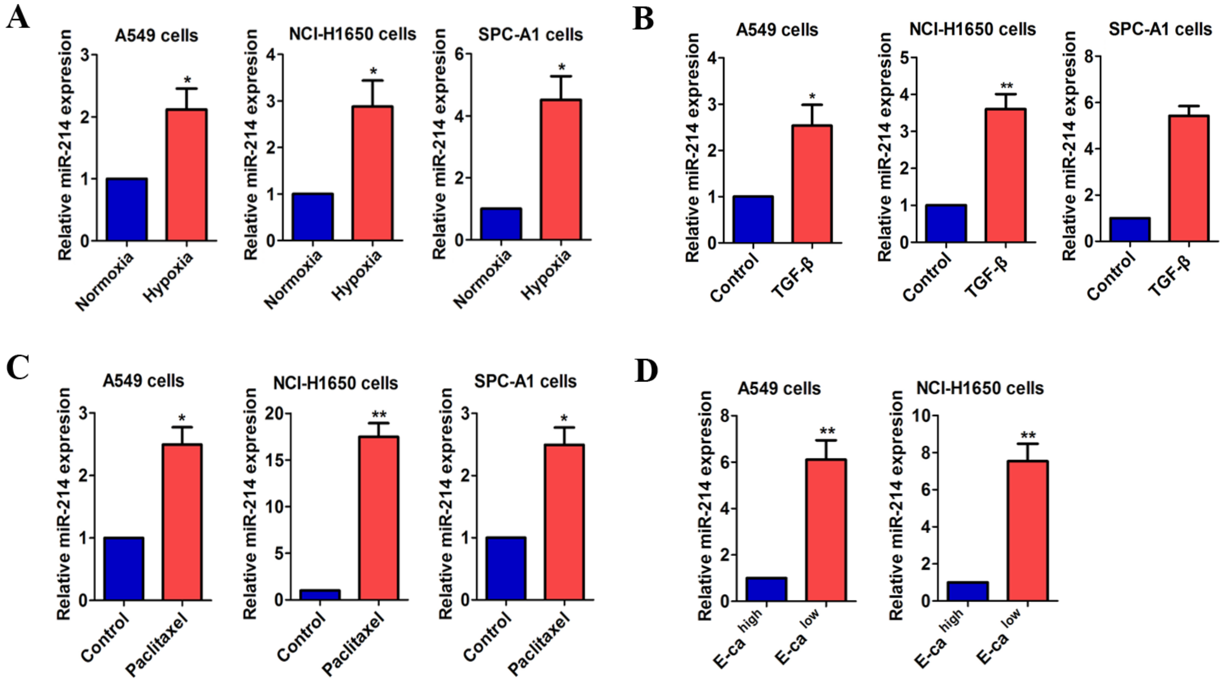

Supplementary Figure S3: A. Fold changes in miR-214 were detected by real-time PCR in three LCCs (A549, NCI-H1650, and SPC-A1) under normoxia (21% O_2) or hypoxia (0.5% O_2) treatment for 24 hours. **B.** miR-214 expression in three LCCs under TGF-β (5 ng/ml) treatment for 7 days. **C.** miR-214 expression in LCCs under paclitaxel (5 ng/ml) treatment for 2 days. **D.** Low E-cadherin-expressing cells (marked as E-ca^{low}) and high expression cells (marked as E-ca^{high}) were sorted by flow cytometry from A549 or NCI-H1650 cells under hypoxia treatment. The fold changes of miR-214 were detected by qRT-PCR. All experiments were performed three times, and the data are expressed as means ± SD. The statistical significance of differences was measured by unpaired student's *t*-test. *$P < 0.05$, **$P < 0.01$.

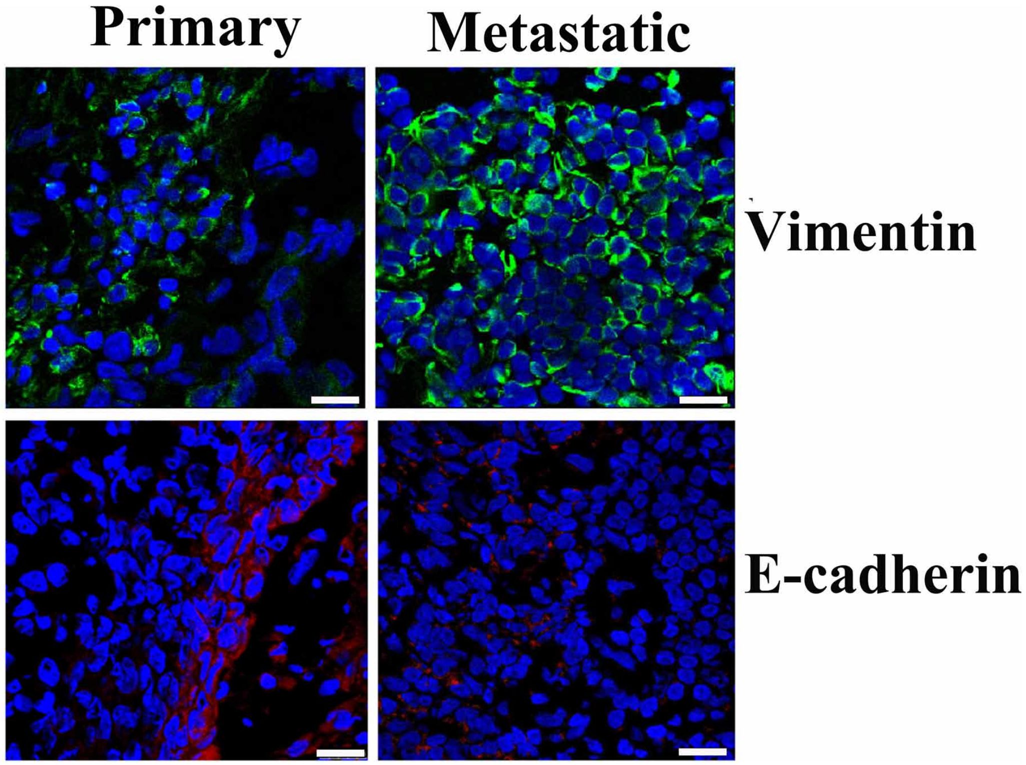

Supplementary Figure S4: Microscopic images showing E-cadherin and vimentin protein levels in primary and metastatic lung tumor pairs (*n* = 10 paired). The nuclei were staining with DAPI. The statistical significance of differences was measured by unpaired student's *t*-test. Scale bar = 25 µm.

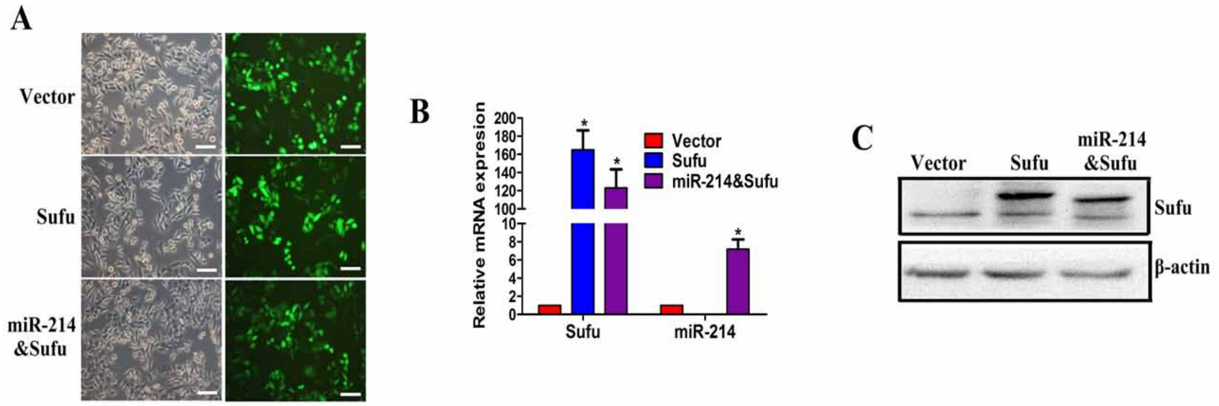

Supplementary Figure S5: A. A morphological image of A549 cells transfected with Sufu-overexpression or both miR-214&Sufu-overexpression or empty lentiviral vectors. Scale bar = 25 µm. **B.** Sufu gene expression levels as tested by qRT-PCR in A549 cells transfected with Sufu or miR-214&Sufu-overexpression virus or the miR-214 expression levels in miR-214&Sufu-overexpression cells. **C.** Sufu protein expression levels was tested by western blot in A549 cells transfected with Sufu-overexpression or miR-214&Sufu-overexpression virus. All experiments were performed three times, and the data are expressed as means ± SD. The statistical significance of differences was measured by unpaired student's *t*-test. $*P < 0.05$, $**P < 0.01$.

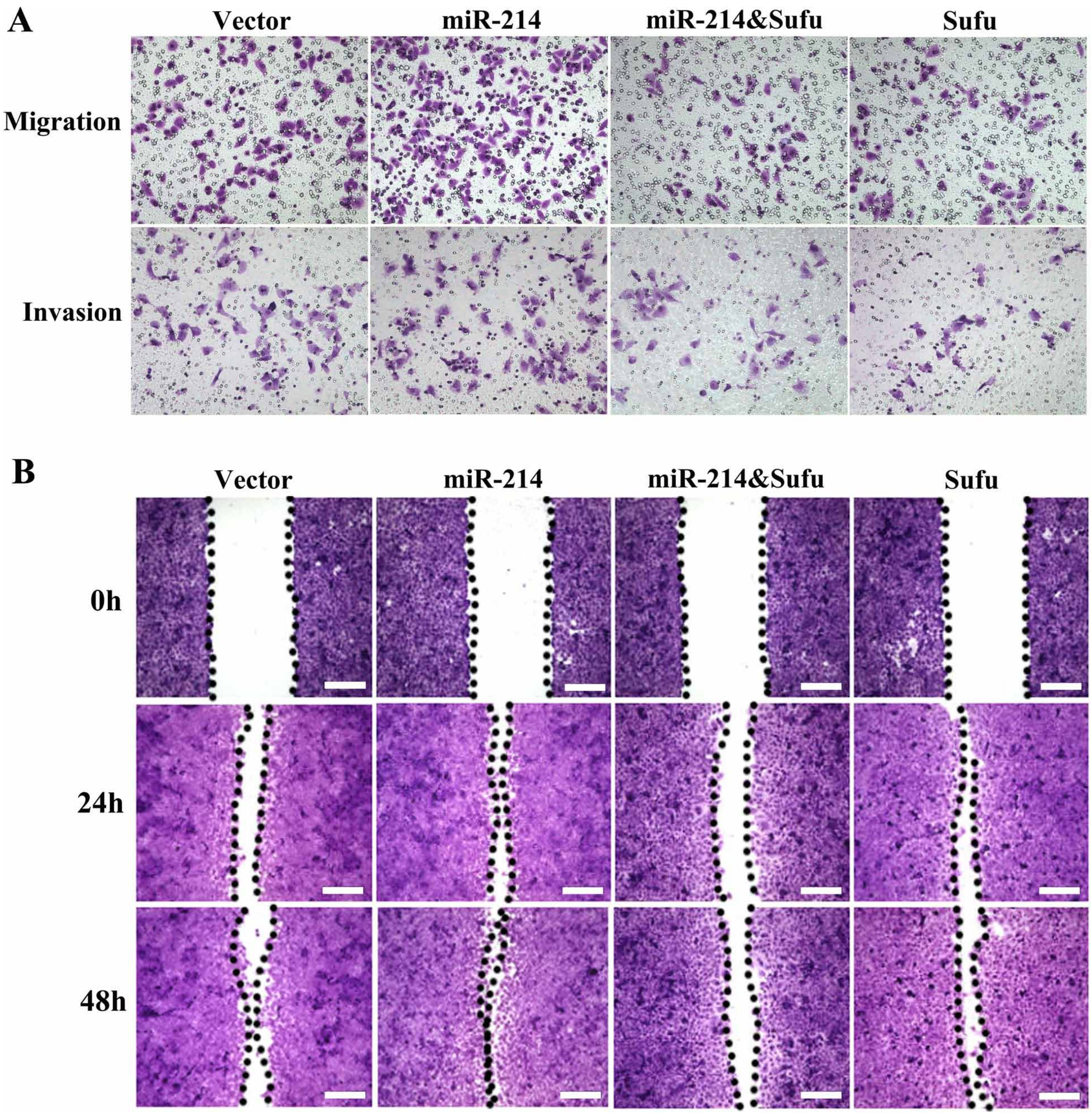

Supplementary Figure S6: A. A morphological image of the invasive and migration capacities of miR-214-over-expressed and/or SUFU-over-expressed A549 cells as assessed by Transwell migration assay and Matrigel invasion assay. **B.** Wound-healing assay images of miR-214-over-expressed and/or SUFU-over-expressed A549 cells at 0, 24, and 48 hours. Scale bar = 25 µm.

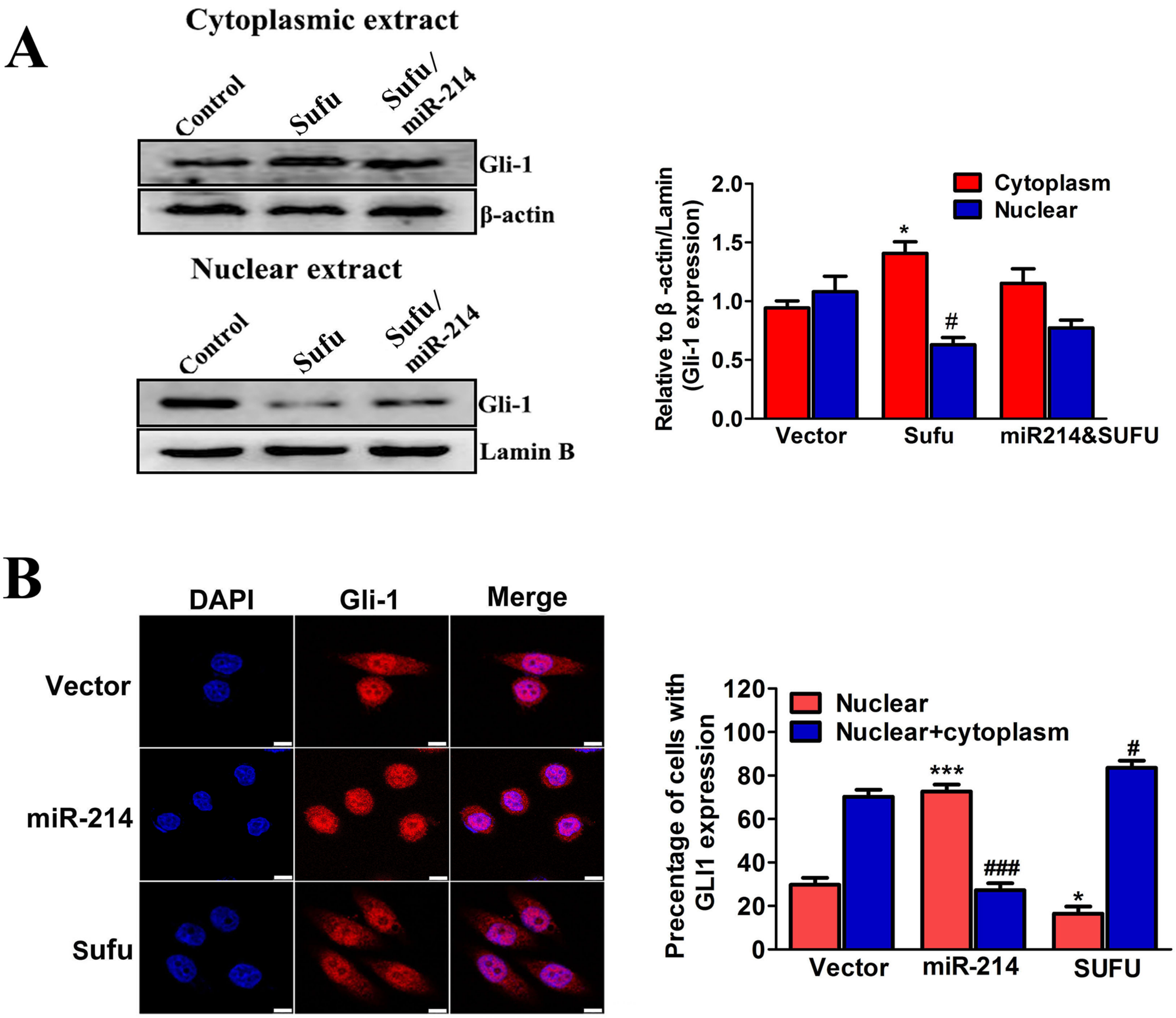

Supplementary Figure S7: A. A western blot analysis of Gli-1 levels in cytoplasmic extract and nuclear extract in the indicated cells. **B.** An immunofluorescence analysis of the Gli-1 nuclear translocation in miR-214-over-expression or Sufu-over-expression A549 cells. Scale bar = 5.0 μm. All experiments were performed three times, and the data are expressed as means ± SD. The statistical significance of differences was measured by unpaired student's *t*-test. $^{*}P$ for nuclear extract group, $^{*}P < 0.05$, $^{**}P < 0.01$, $^{***}P < 0.001$, $^{\#}P$ for nuclear extract group. $^{\#}P < 0.05$, $^{\#\#\#}P < 0.001$.

Supplementary Table S1: Information about the LAC patients

Information about the lung cancer tissues						
ID	Metastasis	Sex	Age	Tumor size mm*mm*mm	Histology	Disease grade
02210660	No	M	44	25*20*16	Adeno	Ia
02745462	No	M	68	30*27*16	Adeno	Ia
01492676	No	F	53	25*24*15	Adeno	Ia
02831476	No	M	53	22*20*16	Adeno	Ia
02303857	No	F	38	20*15*10	Adeno	Ia
00119957	No	F	76	28*27*18	Adeno	Ia
00040714	No	F	54	24*23*15	Adeno	Ia
01938410	No	M	69	30*25*17	Adeno	Ia
01892459	No	M	71	25*24*15	Adeno	Ia
01664226	No	M	75	30*24*15	Adeno	Ia
02210258	No	M	54	25*24*15	Adeno	Ia
03975599	No	F	52	23*20*14	Adeno	Ia
03952421	No	M	46	45*40*30	Adeno	Ib
04163192	No	M	46	30*25*15	Adeno	Ib
03457132	No	M	66	50*45*45	Adeno	Ib
04176656	No	F	65	60*40*30	Adeno	Ib
04127588	No	M	56	25*24*18	Adeno	Ib
04093251	No	F	54	30*25*20	Adeno	Ibx
04157797	Yes	M	54	50*45*40	Adeno	II
102017305	Yes	M	61	45*25*15	Adeno	IIa
04059539	Yes	M	64	40*35*35	Adeno	IIa
04175374	Yes	M	63	40*30*18	Adeno	IIa
03945988	Yes	F	43	18*12*7	Adeno	IIb
03973858	No	M	64	50*30*15	Adeno	IIb
03996058	Yes	M	55	50*50*40	Adeno	IIb
04096772	No	M	61	45*40*30	Adeno	IIb
04176036	No	M	44	85*75*40	Adeno	IIb
03419020	No	F	60	35*34*25	Adeno	IIb
03977769	Yes	M	45	55*50*35	Adeno	IIIa
04034458	Yes	M	50	45*30*30	Adeno	IIIa
04176041	Yes	M	64	20*20*15	Adeno	IIIa
04196680	No	F	58	70*60*56	Adeno	IIIa
04095714	No	F	50	60*55*45	Adeno	IIIa

(*Continued*)

ID	Metastasis	Sex	Age	Tumor size mm*mm*mm	Histology	Disease grade
200220265	Yes	F	45	65*55*50	Adeno	IIIa
03985952	Yes	M	55	65*60*55	Adeno	IV
04096256	Yes	M	65	45*35*30	Adeno	IV
**Information about the paracancerous tissues**						
02210660	No	M	44	25*20*16	Adeno	Ia
02745462	No	M	68	30*27*16	Adeno	Ia
01492676	No	F	53	25*24*15	Adeno	Ia
02831476	No	M	53	22*20*16	Adeno	Ia
03975599	No	F	52	23*20*14	Adeno	Ia
03952421	No	M	46	45*40*30	Adeno	Ib
00137813	No	F	52	30*25*20	Adeno	Ib
03945988	Yes	F	43	18*12*7	Adeno	IIb
03973858	No	M	64	50*30*15	Adeno	IIb
03996058	Yes	M	55	50*50*40	Adeno	IIb
03977769	Yes	M	45	55*50*35	Adeno	IIIa
04034458	Yes	M	50	45*30*30	Adeno	IIIa
02114062	Yes	F	66	65*55*45	Adeno	IV
**Information about the primary and metastatic tissues**						
2484507	Yes	M	66	20*15*15	Adeno	IIa
2640196	Yes	M	55	40*40*30	Adeno	IIb
1170635	Yes	M	60	40*40*50	Adeno	IIb
04176041	Yes	M	64	20*20*15	Adeno	IIIa
1156491	Yes	M	63	40*40*30	Adeno	IIIa
2390076	Yes	F	65	45*40*40	Adeno	IIIa
1117573	Yes	F	55	40*30*20	Adeno	IIIb
2237362	Yes	F	59	38*35*32	Adeno	IV

F: female; M: male; and Adeno: lung adenocarcinoma.

Supplementary Table S2: Primers for selected genes

Gene name	Primers		Product bp
	Sense	Antisense	
E-cadherin	GTCTGTCATGGAAGGTGCT	TACGACGTTAGCCTCGTTC	320
Vimentin	CCACGAAGAGGAAATCCAGG	CAGAGAGGTCAGCAAACTTGG	188
Snail	GAGGCGGTGGCAGACTAG	GACACATCGGTCAGACCAG	159
MMP-9	GGCATCCGGCACCTCTATGGTCC	GCCACTTGTCGGCGATAAGGAAGG	256
Oct-4	AAGCTGCTGAAACAGAAGAGG	ACACGGTTCTCAATGCTAGTC	210
CD326	ATCGTCAATGCCAGTGTA	CTCGCTCAGAGCAGGTTA	288
Nanog	CAGCTGTGTGTACTCAATGATAGATTT	CAACTGGCCGAAGAATAGCAATGGTGT	142
Sufu	CTCCTGACTTGTAACAGGGACC	AGTTGCAAGCAGGGAGAAAA	155
Twist	CGGGAGTCCGCAGTCTTA	GCTTGAGGGTCTGAATCTTG	161
β-actin	TGGAGAAGAGCTATGAGCTGCCTG	GTGCCACCAGACAGCACTGTGTTG	201
